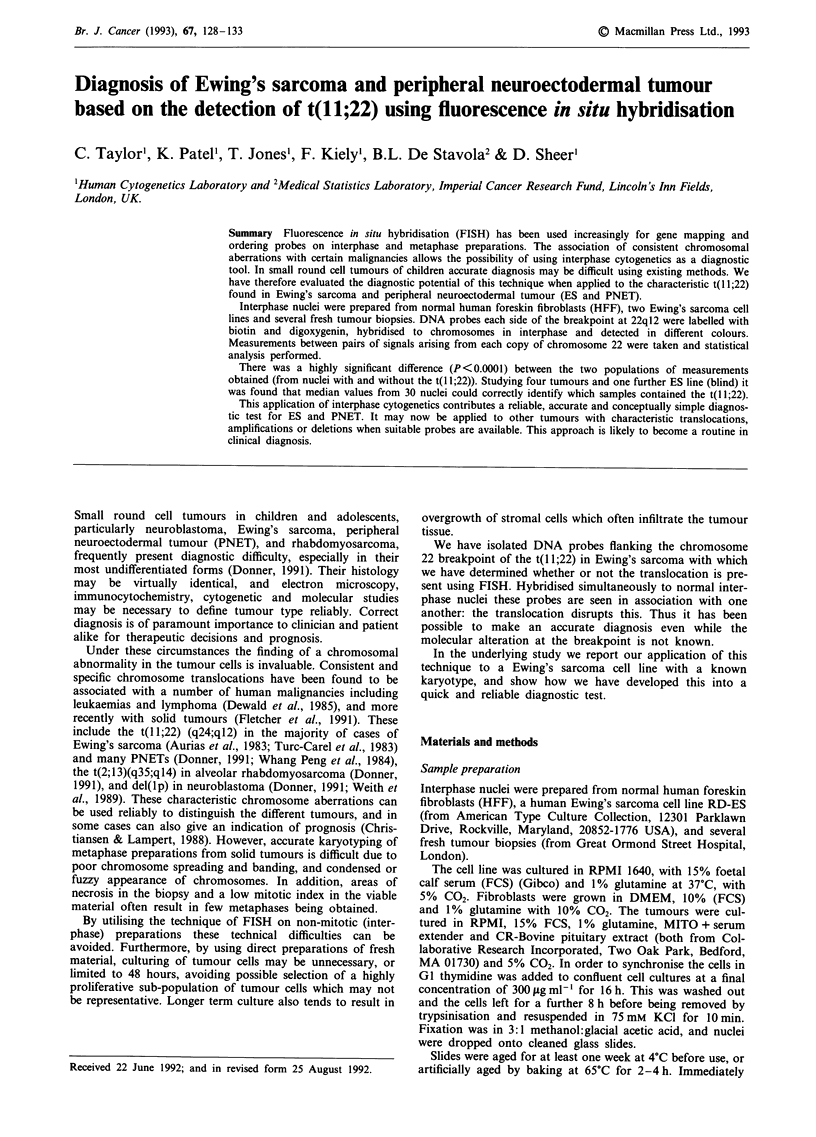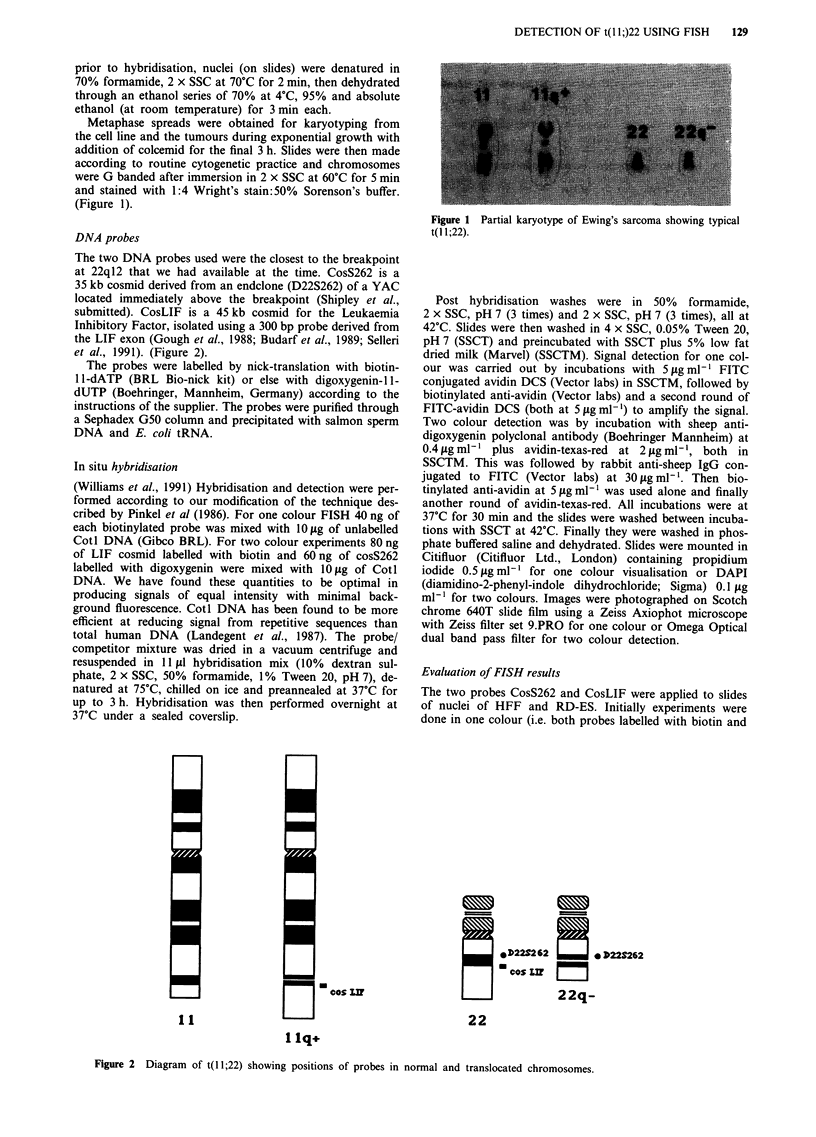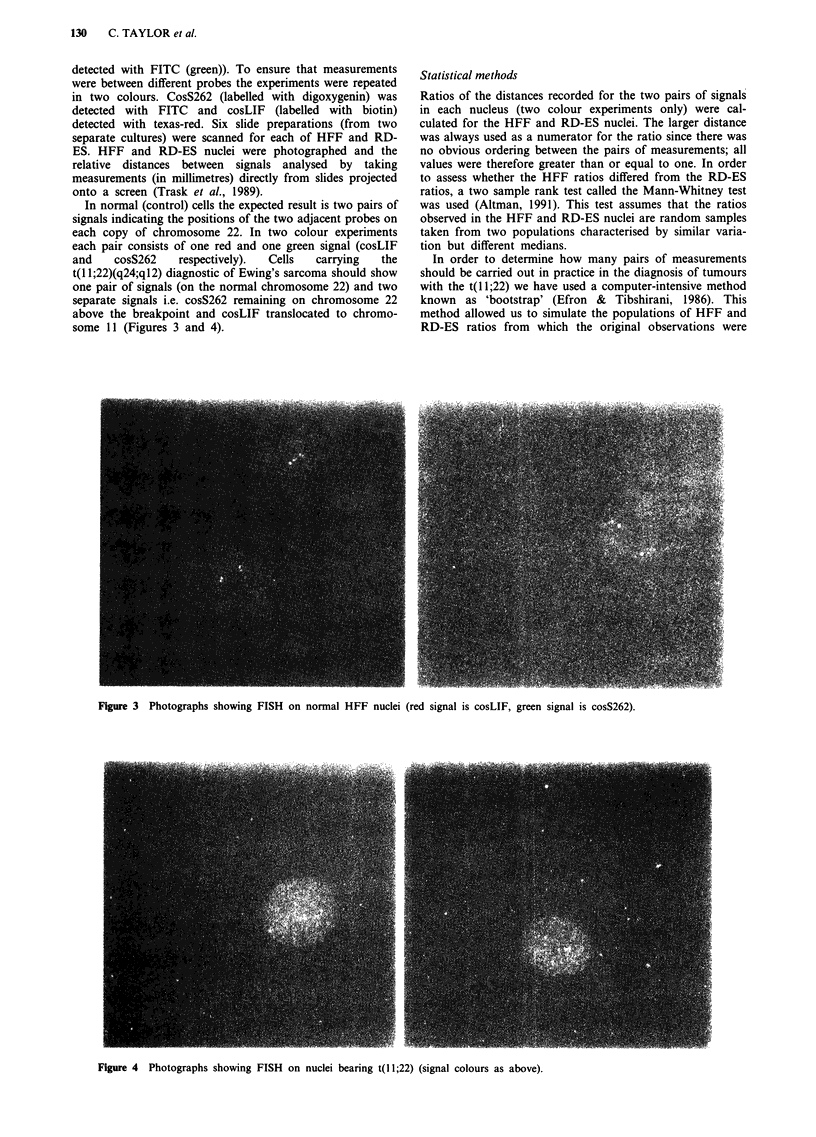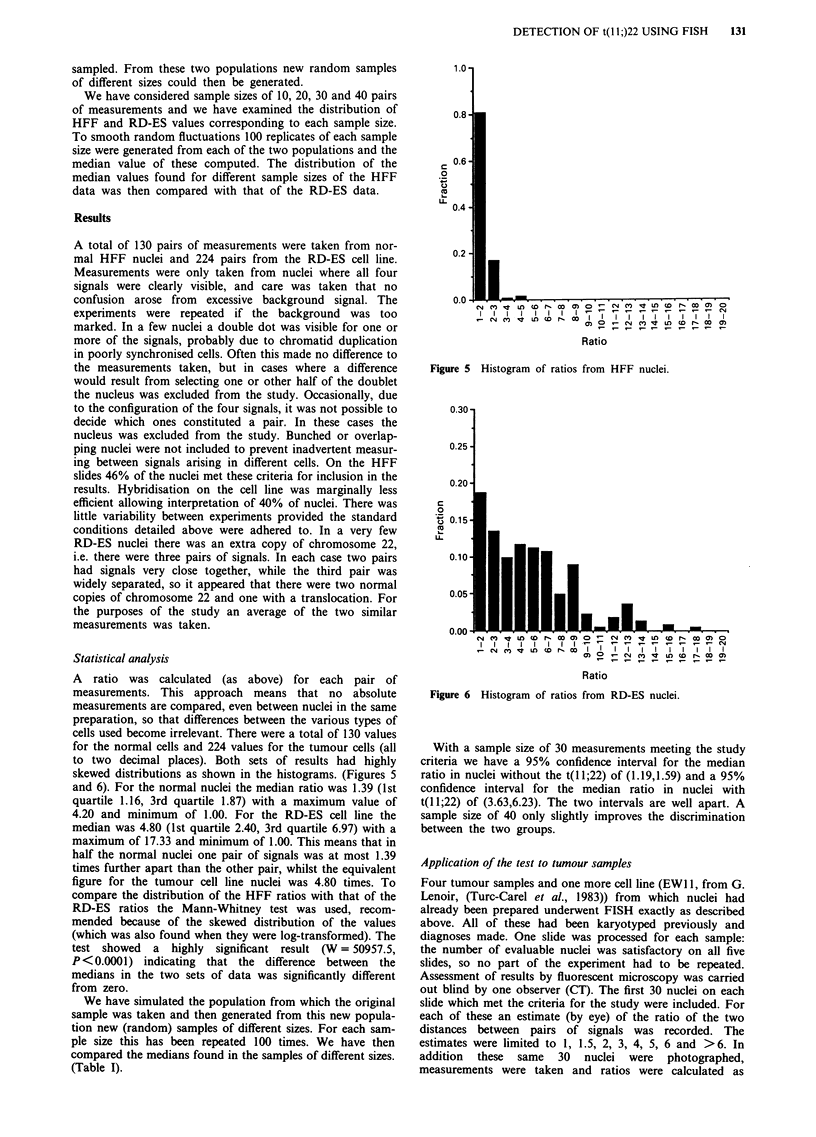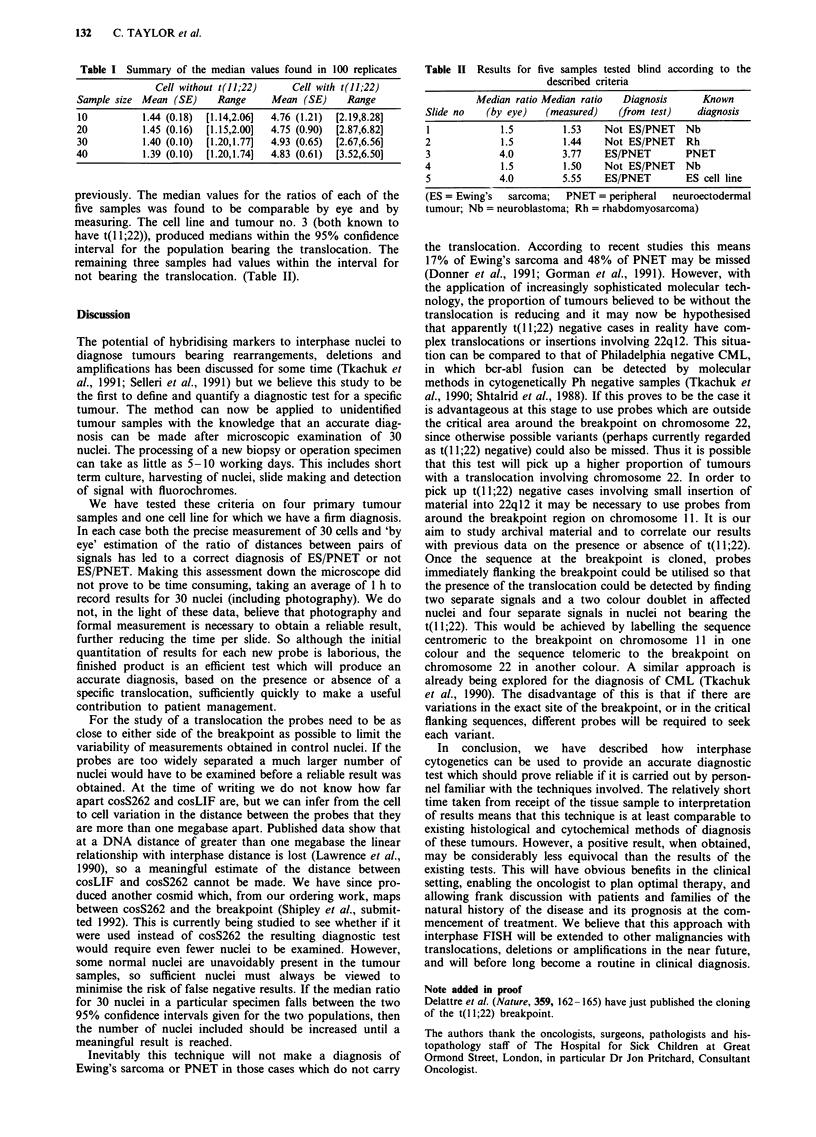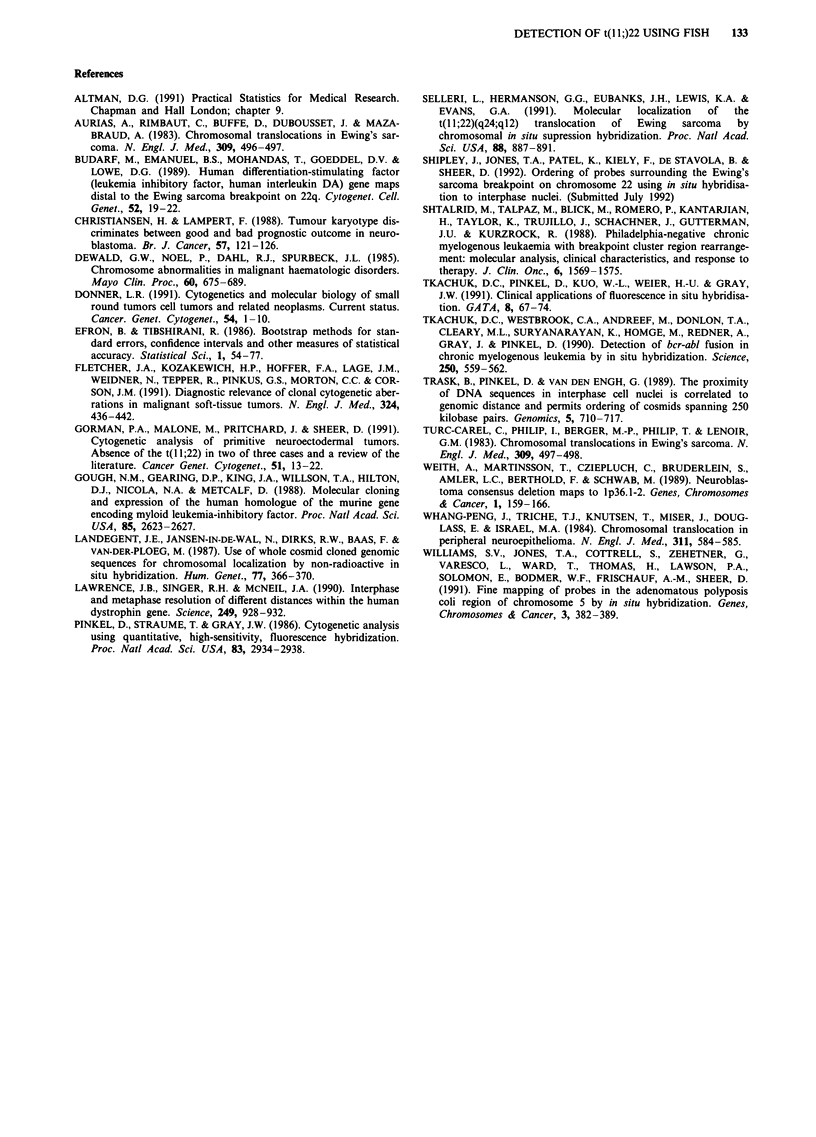# Diagnosis of Ewing's sarcoma and peripheral neuroectodermal tumour based on the detection of t(11;22) using fluorescence in situ hybridisation.

**DOI:** 10.1038/bjc.1993.22

**Published:** 1993-01

**Authors:** C. Taylor, K. Patel, T. Jones, F. Kiely, B. L. De Stavola, D. Sheer

**Affiliations:** Human Cytogenetics Laboratory, Imperial Cancer Research Fund, London, UK.

## Abstract

**Images:**


Br. J. Cancer (1993), 67, 128-133 © Macmillan Press Ltd., 1993

### Diagnosis of Ewing's sarcoma and peripheral neuroectodermal tumour based on the detection of t(11;22) using fluorescence *in situ* hybridisation

C. Taylor[1], K. Patel[1], T. Jones[1], F. Kiely[1], B.L. De Stavola[2] & D. Sheer[1]

[1]*Human Cytogenetics Laboratory and* [2]*Medical Statistics Laboratory, Imperial Cancer Research Fund, Lincoln's Inn Fields, London, UK.*

**Summary** Fluorescence *in situ* hybridisation (FISH) has been used increasingly for gene mapping and ordering probes on interphase and metaphase preparations. The association of consistent chromosomal aberrations with certain malignancies allows the possibility of using interphase cytogenetics as a diagnostic tool. In small round cell tumours of children accurate diagnosis may be difficult using existing methods. We have therefore evaluated the diagnostic potential of this technique when applied to the characteristic t(11;22) found in Ewing's sarcoma and peripheral neuroectodermal tumour (ES and PNET).

Interphase nuclei were prepared from normal human foreskin fibroblasts (HFF), two Ewing's sarcoma cell lines and several fresh tumour biopsies. DNA probes each side of the breakpoint at 22q12 were labelled with biotin and digoxygenin, hybridised to chromosomes in interphase and detected in different colours. Measurements between pairs of signals arising from each copy of chromosome 22 were taken and statistical analysis performed.

There was a highly significant difference ($P < 0.0001$) between the two populations of measurements obtained (from nuclei with and without the t(11;22)). Studying four tumours and one further ES line (blind) it was found that median values from 30 nuclei could correctly identify which samples contained the t(11;22).

This application of interphase cytogenetics contributes a reliable, accurate and conceptually simple diagnostic test for ES and PNET. It may now be applied to other tumours with characteristic translocations, amplifications or deletions when suitable probes are available. This approach is likely to become a routine in clinical diagnosis.

Small round cell tumours in children and adolescents, particularly neuroblastoma, Ewing's sarcoma, peripheral neuroectodermal tumour (PNET), and rhabdomyosarcoma, frequently present diagnostic difficulty, especially in their most undifferentiated forms (Donner, 1991). Their histology may be virtually identical, and electron microscopy, immunocytochemistry, cytogenetic and molecular studies may be necessary to define tumour type reliably. Correct diagnosis is of paramount importance to clinician and patient alike for therapeutic decisions and prognosis.

Under these circumstances the finding of a chromosomal abnormality in the tumour cells is invaluable. Consistent and specific chromosome translocations have been found to be associated with a number of human malignancies including leukaemias and lymphoma (Dewald *et al.*, 1985), and more recently with solid tumours (Fletcher *et al.*, 1991). These include the t(11;22) (q24;q12) in the majority of cases of Ewing's sarcoma (Aurias *et al.*, 1983; Turc-Carel *et al.*, 1983) and many PNETs (Donner, 1991; Whang Peng *et al.*, 1984), the t(2;13)(q35;q14) in alveolar rhabdomyosarcoma (Donner, 1991), and del(1p) in neuroblastoma (Donner, 1991; Weith *et al.*, 1989). These characteristic chromosome aberrations can be used reliably to distinguish the different tumours, and in some cases can also give an indication of prognosis (Christiansen & Lampert, 1988). However, accurate karyotyping of metaphase preparations from solid tumours is difficult due to poor chromosome spreading and banding, and condensed or fuzzy appearance of chromosomes. In addition, areas of necrosis in the biopsy and a low mitotic index in the viable material often result in few metaphases being obtained.

By utilising the technique of FISH on non-mitotic (interphase) preparations these technical difficulties can be avoided. Furthermore, by using direct preparations of fresh material, culturing of tumour cells may be unnecessary, or limited to 48 hours, avoiding possible selection of a highly proliferative sub-population of tumour cells which may not be representative. Longer term culture also tends to result in overgrowth of stromal cells which often infiltrate the tumour tissue.

We have isolated DNA probes flanking the chromosome 22 breakpoint of the t(11;22) in Ewing's sarcoma with which we have determined whether or not the translocation is present using FISH. Hybridised simultaneously to normal interphase nuclei these probes are seen in association with one another: the translocation disrupts this. Thus it has been possible to make an accurate diagnosis even while the molecular alteration at the breakpoint is not known.

In the underlying study we report our application of this technique to a Ewing's sarcoma cell line with a known karyotype, and show how we have developed this into a quick and reliable diagnostic test.

### Materials and methods

### *Sample preparation*

Interphase nuclei were prepared from normal human foreskin fibroblasts (HFF), a human Ewing's sarcoma cell line RD-ES (from American Type Culture Collection, 12301 Parklawn Drive, Rockville, Maryland, 20852-1776 USA), and several fresh tumour biopsies (from Great Ormond Street Hospital, London).

The cell line was cultured in RPMI 1640, with 15% foetal calf serum (FCS) (Gibco) and 1% glutamine at 37°C, with 5% $CO_2$. Fibroblasts were grown in DMEM, 10% (FCS) and 1% glutamine with 10% $CO_2$. The tumours were cultured in RPMI, 15% FCS, 1% glutamine, MITO + serum extender and CR-Bovine pituitary extract (both from Collaborative Research Incorporated, Two Oak Park, Bedford, MA 01730) and 5% $CO_2$. In order to synchronise the cells in G1 thymidine was added to confluent cell cultures at a final concentration of 300 $\mu g\ ml^{-1}$ for 16 h. This was washed out and the cells left for a further 8 h before being removed by trypsinisation and resuspended in 75 mM KCl for 10 min. Fixation was in 3:1 methanol:glacial acetic acid, and nuclei were dropped onto cleaned glass slides.

Slides were aged for at least one week at 4°C before use, or artificially aged by baking at 65°C for 2–4 h. Immediately

Received 22 June 1992; and in revised form 25 August 1992.

prior to hybridisation, nuclei (on slides) were denatured in 70% formamide, 2 × SSC at 70°C for 2 min, then dehydrated through an ethanol series of 70% at 4°C, 95% and absolute ethanol (at room temperature) for 3 min each.

Metaphase spreads were obtained for karyotyping from the cell line and the tumours during exponential growth with addition of colcemid for the final 3 h. Slides were then made according to routine cytogenetic practice and chromosomes were G banded after immersion in 2 × SSC at 60°C for 5 min and stained with 1:4 Wright's stain:50% Sorenson's buffer. (Figure 1).

**Figure 1** Partial karyotype of Ewing's sarcoma showing typical t(11;22).

*DNA probes*

The two DNA probes used were the closest to the breakpoint at 22q12 that we had available at the time. CosS262 is a 35 kb cosmid derived from an endclone (D22S262) of a YAC located immediately above the breakpoint (Shipley *et al.*, submitted). CosLIF is a 45 kb cosmid for the Leukaemia Inhibitory Factor, isolated using a 300 bp probe derived from the LIF exon (Gough *et al.*, 1988; Budarf *et al.*, 1989; Selleri *et al.*, 1991). (Figure 2).

The probes were labelled by nick-translation with biotin-11-dATP (BRL Bio-nick kit) or else with digoxygenin-11-dUTP (Boehringer, Mannheim, Germany) according to the instructions of the supplier. The probes were purified through a Sephadex G50 column and precipitated with salmon sperm DNA and *E. coli* tRNA.

In situ *hybridisation*

(Williams *et al.*, 1991) Hybridisation and detection were performed according to our modification of the technique described by Pinkel *et al* (1986). For one colour FISH 40 ng of each biotinylated probe was mixed with 10 μg of unlabelled Cot1 DNA (Gibco BRL). For two colour experiments 80 ng of LIF cosmid labelled with biotin and 60 ng of cosS262 labelled with digoxygenin were mixed with 10 μg of Cot1 DNA. We have found these quantities to be optimal in producing signals of equal intensity with minimal background fluorescence. Cot1 DNA has been found to be more efficient at reducing signal from repetitive sequences than total human DNA (Landegent *et al.*, 1987). The probe/competitor mixture was dried in a vacuum centrifuge and resuspended in 11 μl hybridisation mix (10% dextran sulphate, 2 × SSC, 50% formamide, 1% Tween 20, pH 7), denatured at 75°C, chilled on ice and preannealed at 37°C for up to 3 h. Hybridisation was then performed overnight at 37°C under a sealed coverslip.

Post hybridisation washes were in 50% formamide, 2 × SSC, pH 7 (3 times) and 2 × SSC, pH 7 (3 times), all at 42°C. Slides were then washed in 4 × SSC, 0.05% Tween 20, pH 7 (SSCT) and preincubated with SSCT plus 5% low fat dried milk (Marvel) (SSCTM). Signal detection for one colour was carried out by incubations with 5 $\mu g\, ml^{-1}$ FITC conjugated avidin DCS (Vector labs) in SSCTM, followed by biotinylated anti-avidin (Vector labs) and a second round of FITC-avidin DCS (both at 5 $\mu g\, ml^{-1}$) to amplify the signal. Two colour detection was by incubation with sheep anti-digoxygenin polyclonal antibody (Boehringer Mannheim) at 0.4 $\mu g\, ml^{-1}$ plus avidin-texas-red at 2 $\mu g\, ml^{-1}$, both in SSCTM. This was followed by rabbit anti-sheep IgG conjugated to FITC (Vector labs) at 30 $\mu g\, ml^{-1}$. Then biotinylated anti-avidin at 5 $\mu g\, ml^{-1}$ was used alone and finally another round of avidin-texas-red. All incubations were at 37°C for 30 min and the slides were washed between incubations with SSCT at 42°C. Finally they were washed in phosphate buffered saline and dehydrated. Slides were mounted in Citifluor (Citifluor Ltd., London) containing propidium iodide 0.5 $\mu g\, ml^{-1}$ for one colour visualisation or DAPI (diamidino-2-phenyl-indole dihydrochloride; Sigma) 0.1 μg $ml^{-1}$ for two colours. Images were photographed on Scotch chrome 640T slide film using a Zeiss Axiophot microscope with Zeiss filter set 9.PRO for one colour or Omega Optical dual band pass filter for two colour detection.

*Evaluation of FISH results*

The two probes CosS262 and CosLIF were applied to slides of nuclei of HFF and RD-ES. Initially experiments were done in one colour (i.e. both probes labelled with biotin and

**Figure 2** Diagram of t(11;22) showing positions of probes in normal and translocated chromosomes.

detected with FITC (green)). To ensure that measurements were between different probes the experiments were repeated in two colours. CosS262 (labelled with digoxygenin) was detected with FITC and cosLIF (labelled with biotin) detected with texas-red. Six slide preparations (from two separate cultures) were scanned for each of HFF and RD-ES. HFF and RD-ES nuclei were photographed and the relative distances between signals analysed by taking measurements (in millimetres) directly from slides projected onto a screen (Trask *et al.*, 1989).

In normal (control) cells the expected result is two pairs of signals indicating the positions of the two adjacent probes on each copy of chromosome 22. In two colour experiments each pair consists of one red and one green signal (cosLIF and cosS262 respectively). Cells carrying the t(11;22)(q24;q12) diagnostic of Ewing's sarcoma should show one pair of signals (on the normal chromosome 22) and two separate signals i.e. cosS262 remaining on chromosome 22 above the breakpoint and cosLIF translocated to chromosome 11 (Figures 3 and 4).

*Statistical methods*

Ratios of the distances recorded for the two pairs of signals in each nucleus (two colour experiments only) were calculated for the HFF and RD-ES nuclei. The larger distance was always used as a numerator for the ratio since there was no obvious ordering between the pairs of measurements; all values were therefore greater than or equal to one. In order to assess whether the HFF ratios differed from the RD-ES ratios, a two sample rank test called the Mann-Whitney test was used (Altman, 1991). This test assumes that the ratios observed in the HFF and RD-ES nuclei are random samples taken from two populations characterised by similar variation but different medians.

In order to determine how many pairs of measurements should be carried out in practice in the diagnosis of tumours with the t(11;22) we have used a computer-intensive method known as 'bootstrap' (Efron & Tibshirani, 1986). This method allowed us to simulate the populations of HFF and RD-ES ratios from which the original observations were

**Figure 3** Photographs showing FISH on normal HFF nuclei (red signal is cosLIF, green signal is cosS262).

**Figure 4** Photographs showing FISH on nuclei bearing t(11;22) (signal colours as above).

sampled. From these two populations new random samples of different sizes could then be generated.

We have considered sample sizes of 10, 20, 30 and 40 pairs of measurements and we have examined the distribution of HFF and RD-ES values corresponding to each sample size. To smooth random fluctuations 100 replicates of each sample size were generated from each of the two populations and the median value of these computed. The distribution of the median values found for different sample sizes of the HFF data was then compared with that of the RD-ES data.

### Results

A total of 130 pairs of measurements were taken from normal HFF nuclei and 224 pairs from the RD-ES cell line. Measurements were only taken from nuclei where all four signals were clearly visible, and care was taken that no confusion arose from excessive background signal. The experiments were repeated if the background was too marked. In a few nuclei a double dot was visible for one or more of the signals, probably due to chromatid duplication in poorly synchronised cells. Often this made no difference to the measurements taken, but in cases where a difference would result from selecting one or other half of the doublet the nucleus was excluded from the study. Occasionally, due to the configuration of the four signals, it was not possible to decide which ones constituted a pair. In these cases the nucleus was excluded from the study. Bunched or overlapping nuclei were not included to prevent inadvertent measuring between signals arising in different cells. On the HFF slides 46% of the nuclei met these criteria for inclusion in the results. Hybridisation on the cell line was marginally less efficient allowing interpretation of 40% of nuclei. There was little variability between experiments provided the standard conditions detailed above were adhered to. In a very few RD-ES nuclei there was an extra copy of chromosome 22, i.e. there were three pairs of signals. In each case two pairs had signals very close together, while the third pair was widely separated, so it appeared that there were two normal copies of chromosome 22 and one with a translocation. For the purposes of the study an average of the two similar measurements was taken.

### *Statistical analysis*

A ratio was calculated (as above) for each pair of measurements. This approach means that no absolute measurements are compared, even between nuclei in the same preparation, so that differences between the various types of cells used become irrelevant. There were a total of 130 values for the normal cells and 224 values for the tumour cells (all to two decimal places). Both sets of results had highly skewed distributions as shown in the histograms. (Figures 5 and 6). For the normal nuclei the median ratio was 1.39 (1st quartile 1.16, 3rd quartile 1.87) with a maximum value of 4.20 and minimum of 1.00. For the RD-ES cell line the median was 4.80 (1st quartile 2.40, 3rd quartile 6.97) with a maximum of 17.33 and minimum of 1.00. This means that in half the normal nuclei one pair of signals was at most 1.39 times further apart than the other pair, whilst the equivalent figure for the tumour cell line nuclei was 4.80 times. To compare the distribution of the HFF ratios with that of the RD-ES ratios the Mann-Whitney test was used, recommended because of the skewed distribution of the values (which was also found when they were log-transformed). The test showed a highly significant result ($W = 50957.5$, $P < 0.0001$) indicating that the difference between the medians in the two sets of data was significantly different from zero.

We have simulated the population from which the original sample was taken and then generated from this new population new (random) samples of different sizes. For each sample size this has been repeated 100 times. We have then compared the medians found in the samples of different sizes. (Table I).

**Figure 5** Histogram of ratios from HFF nuclei.

**Figure 6** Histogram of ratios from RD-ES nuclei.

With a sample size of 30 measurements meeting the study criteria we have a 95% confidence interval for the median ratio in nuclei without the t(11;22) of (1.19,1.59) and a 95% confidence interval for the median ratio in nuclei with t(11;22) of (3.63,6.23). The two intervals are well apart. A sample size of 40 only slightly improves the discrimination between the two groups.

### *Application of the test to tumour samples*

Four tumour samples and one more cell line (EW11, from G. Lenoir, (Turc-Carel *et al.*, 1983)) from which nuclei had already been prepared underwent FISH exactly as described above. All of these had been karyotyped previously and diagnoses made. One slide was processed for each sample: the number of evaluable nuclei was satisfactory on all five slides, so no part of the experiment had to be repeated. Assessment of results by fluorescent microscopy was carried out blind by one observer (CT). The first 30 nuclei on each slide which met the criteria for the study were included. For each of these an estimate (by eye) of the ratio of the two distances between pairs of signals was recorded. The estimates were limited to 1, 1.5, 2, 3, 4, 5, 6 and >6. In addition these same 30 nuclei were photographed, measurements were taken and ratios were calculated as

**Table I** Summary of the median values found in 100 replicates

| | *Cell without t(11;22)* | | *Cell with t(11;22)* | |
|---|---|---|---|---|
| *Sample size* | *Mean (SE)* | *Range* | *Mean (SE)* | *Range* |
| 10 | 1.44 (0.18) | [1.14,2.06] | 4.76 (1.21) | [2.19,8.28] |
| 20 | 1.45 (0.16) | [1.15,2.00] | 4.75 (0.90) | [2.87,6.82] |
| 30 | 1.40 (0.10) | [1.20,1.77] | 4.93 (0.65) | [2.67,6.56] |
| 40 | 1.39 (0.10) | [1.20,1.74] | 4.83 (0.61) | [3.52,6.50] |

**Table II** Results for five samples tested blind according to the described criteria

| *Slide no* | *Median ratio (by eye)* | *Median ratio (measured)* | *Diagnosis (from test)* | *Known diagnosis* |
|---|---|---|---|---|
| 1 | 1.5 | 1.53 | Not ES/PNET | Nb |
| 2 | 1.5 | 1.44 | Not ES/PNET | Rh |
| 3 | 4.0 | 3.77 | ES/PNET | PNET |
| 4 | 1.5 | 1.50 | Not ES/PNET | Nb |
| 5 | 4.0 | 5.55 | ES/PNET | ES cell line |

(ES = Ewing's sarcoma; PNET = peripheral neuroectodermal tumour; Nb = neuroblastoma; Rh = rhabdomyosarcoma)

previously. The median values for the ratios of each of the five samples was found to be comparable by eye and by measuring. The cell line and tumour no. 3 (both known to have t(11;22)), produced medians within the 95% confidence interval for the population bearing the translocation. The remaining three samples had values within the interval for not bearing the translocation. (Table II).

### Discussion

The potential of hybridising markers to interphase nuclei to diagnose tumours bearing rearrangements, deletions and amplifications has been discussed for some time (Tkachuk *et al.*, 1991; Selleri *et al.*, 1991) but we believe this study to be the first to define and quantify a diagnostic test for a specific tumour. The method can now be applied to unidentified tumour samples with the knowledge that an accurate diagnosis can be made after microscopic examination of 30 nuclei. The processing of a new biopsy or operation specimen can take as little as 5–10 working days. This includes short term culture, harvesting of nuclei, slide making and detection of signal with fluorochromes.

We have tested these criteria on four primary tumour samples and one cell line for which we have a firm diagnosis. In each case both the precise measurement of 30 cells and 'by eye' estimation of the ratio of distances between pairs of signals has led to a correct diagnosis of ES/PNET or not ES/PNET. Making this assessment down the microscope did not prove to be time consuming, taking an average of 1 h to record results for 30 nuclei (including photography). We do not, in the light of these data, believe that photography and formal measurement is necessary to obtain a reliable result, further reducing the time per slide. So although the initial quantitation of results for each new probe is laborious, the finished product is an efficient test which will produce an accurate diagnosis, based on the presence or absence of a specific translocation, sufficiently quickly to make a useful contribution to patient management.

For the study of a translocation the probes need to be as close to either side of the breakpoint as possible to limit the variability of measurements obtained in control nuclei. If the probes are too widely separated a much larger number of nuclei would have to be examined before a reliable result was obtained. At the time of writing we do not know how far apart cosS262 and cosLIF are, but we can infer from the cell to cell variation in the distance between the probes that they are more than one megabase apart. Published data show that at a DNA distance of greater than one megabase the linear relationship with interphase distance is lost (Lawrence *et al.*, 1990), so a meaningful estimate of the distance between cosLIF and cosS262 cannot be made. We have since produced another cosmid which, from our ordering work, maps between cosS262 and the breakpoint (Shipley *et al.*, submitted 1992). This is currently being studied to see whether if it were used instead of cosS262 the resulting diagnostic test would require even fewer nuclei to be examined. However, some normal nuclei are unavoidably present in the tumour samples, so sufficient nuclei must always be viewed to minimise the risk of false negative results. If the median ratio for 30 nuclei in a particular specimen falls between the two 95% confidence intervals given for the two populations, then the number of nuclei included should be increased until a meaningful result is reached.

Inevitably this technique will not make a diagnosis of Ewing's sarcoma or PNET in those cases which do not carry the translocation. According to recent studies this means 17% of Ewing's sarcoma and 48% of PNET may be missed (Donner *et al.*, 1991; Gorman *et al.*, 1991). However, with the application of increasingly sophisticated molecular technology, the proportion of tumours believed to be without the translocation is reducing and it may now be hypothesised that apparently t(11;22) negative cases in reality have complex translocations or insertions involving 22q12. This situation can be compared to that of Philadelphia negative CML, in which bcr-abl fusion can be detected by molecular methods in cytogenetically Ph negative samples (Tkachuk *et al.*, 1990; Shtalrid *et al.*, 1988). If this proves to be the case it is advantageous at this stage to use probes which are outside the critical area around the breakpoint on chromosome 22, since otherwise possible variants (perhaps currently regarded as t(11;22) negative) could also be missed. Thus it is possible that this test will pick up a higher proportion of tumours with a translocation involving chromosome 22. In order to pick up t(11;22) negative cases involving small insertion of material into 22q12 it may be necessary to use probes from around the breakpoint region on chromosome 11. It is our aim to study archival material and to correlate our results with previous data on the presence or absence of t(11;22). Once the sequence at the breakpoint is cloned, probes immediately flanking the breakpoint could be utilised so that the presence of the translocation could be detected by finding two separate signals and a two colour doublet in affected nuclei and four separate signals in nuclei not bearing the t(11;22). This would be achieved by labelling the sequence centromeric to the breakpoint on chromosome 11 in one colour and the sequence telomeric to the breakpoint on chromosome 22 in another colour. A similar approach is already being explored for the diagnosis of CML (Tkachuk *et al.*, 1990). The disadvantage of this is that if there are variations in the exact site of the breakpoint, or in the critical flanking sequences, different probes will be required to seek each variant.

In conclusion, we have described how interphase cytogenetics can be used to provide an accurate diagnostic test which should prove reliable if it is carried out by personnel familiar with the techniques involved. The relatively short time taken from receipt of the tissue sample to interpretation of results means that this technique is at least comparable to existing histological and cytochemical methods of diagnosis of these tumours. However, a positive result, when obtained, may be considerably less equivocal than the results of the existing tests. This will have obvious benefits in the clinical setting, enabling the oncologist to plan optimal therapy, and allowing frank discussion with patients and families of the natural history of the disease and its prognosis at the commencement of treatment. We believe that this approach with interphase FISH will be extended to other malignancies with translocations, deletions or amplifications in the near future, and will before long become a routine in clinical diagnosis.

**Note added in proof**

Delattre *et al.* (*Nature*, **359**, 162–165) have just published the cloning of the t(11;22) breakpoint.

The authors thank the oncologists, surgeons, pathologists and histopathology staff of The Hospital for Sick Children at Great Ormond Street, London, in particular Dr Jon Pritchard, Consultant Oncologist.

**References**

ALTMAN, D.G. (1991) Practical Statistics for Medical Research. Chapman and Hall London; chapter 9.

AURIAS, A., RIMBAUT, C., BUFFE, D., DUBOUSSET, J. & MAZABRAUD, A. (1983). Chromosomal translocations in Ewing's sarcoma. *N. Engl. J. Med.*, **309**, 496–497.

BUDARF, M., EMANUEL, B.S., MOHANDAS, T., GOEDDEL, D.V. & LOWE, D.G. (1989). Human differentiation-stimulating factor (leukemia inhibitory factor, human interleukin DA) gene maps distal to the Ewing sarcoma breakpoint on 22q. *Cytogenet. Cell. Genet.*, **52**, 19–22.

CHRISTIANSEN, H. & LAMPERT, F. (1988). Tumour karyotype discriminates between good and bad prognostic outcome in neuroblastoma. *Br. J. Cancer*, **57**, 121–126.

DEWALD, G.W., NOEL, P., DAHL, R.J., SPURBECK, J.L. (1985). Chromosome abnormalities in malignant haematologic disorders. *Mayo Clin. Proc.*, **60**, 675–689.

DONNER, L.R. (1991). Cytogenetics and molecular biology of small round tumors cell tumors and related neoplasms. Current status. *Cancer. Genet. Cytogenet.*, **54**, 1–10.

EFRON, B. & TIBSHIRANI, R. (1986). Bootstrap methods for standard errors, confidence intervals and other measures of statistical accuracy. *Statistical Sci.*, **1**, 54–77.

FLETCHER, J.A., KOZAKEWICH, H.P., HOFFER, F.A., LAGE, J.M., WEIDNER, N., TEPPER, R., PINKUS, G.S., MORTON, C.C. & CORSON, J.M. (1991). Diagnostic relevance of clonal cytogenetic aberrations in malignant soft-tissue tumors. *N. Engl. J. Med.*, **324**, 436–442.

GORMAN, P.A., MALONE, M., PRITCHARD, J. & SHEER, D. (1991). Cytogenetic analysis of primitive neuroectodermal tumors. Absence of the t(11;22) in two of three cases and a review of the literature. *Cancer Genet. Cytogenet.*, **51**, 13–22.

GOUGH, N.M., GEARING, D.P., KING, J.A., WILLSON, T.A., HILTON, D.J., NICOLA, N.A. & METCALF, D. (1988). Molecular cloning and expression of the human homologue of the murine gene encoding myloid leukemia-inhibitory factor. *Proc. Natl Acad. Sci. USA*, **85**, 2623–2627.

LANDEGENT, J.E., JANSEN-IN-DE-WAL, N., DIRKS, R.W., BAAS, F. & VAN-DER-PLOEG, M. (1987). Use of whole cosmid cloned genomic sequences for chromosomal localization by non-radioactive in situ hybridization. *Hum. Genet.*, **77**, 366–370.

LAWRENCE, J.B., SINGER, R.H. & MCNEIL, J.A. (1990). Interphase and metaphase resolution of different distances within the human dystrophin gene. *Science*, **249**, 928–932.

PINKEL, D., STRAUME, T. & GRAY, J.W. (1986). Cytogenetic analysis using quantitative, high-sensitivity, fluorescence hybridization. *Proc. Natl Acad. Sci. USA*, **83**, 2934–2938.

SELLERI, L., HERMANSON, G.G., EUBANKS, J.H., LEWIS, K.A. & EVANS, G.A. (1991). Molecular localization of the t(11;22)(q24;q12) translocation of Ewing sarcoma by chromosomal *in situ* supression hybridization. *Proc. Natl Acad. Sci. USA*, **88**, 887–891.

SHIPLEY, J., JONES, T.A., PATEL, K., KIELY, F., DE STAVOLA, B. & SHEER, D. (1992). Ordering of probes surrounding the Ewing's sarcoma breakpoint on chromosome 22 using *in situ* hybridisation to interphase nuclei. (Submitted July 1992)

SHTALRID, M., TALPAZ, M., BLICK, M., ROMERO, P., KANTARJIAN, H., TAYLOR, K., TRUJILLO, J., SCHACHNER, J., GUTTERMAN, J.U. & KURZROCK, R. (1988). Philadelphia-negative chronic myelogenous leukaemia with breakpoint cluster region rearrangement: molecular analysis, clinical characteristics, and response to therapy. *J. Clin. Onc.*, **6**, 1569–1575.

TKACHUK, D.C., PINKEL, D., KUO, W.-L., WEIER, H.-U. & GRAY, J.W. (1991). Clinical applications of fluorescence in situ hybridisation. *GATA*, **8**, 67–74.

TKACHUK, D.C., WESTBROOK, C.A., ANDREEF, M., DONLON, T.A., CLEARY, M.L., SURYANARAYAN, K., HOMGE, M., REDNER, A., GRAY, J. & PINKEL, D. (1990). Detection of *bcr-abl* fusion in chronic myelogenous leukemia by in situ hybridization. *Science*, **250**, 559–562.

TRASK, B., PINKEL, D. & VAN DEN ENGH, G. (1989). The proximity of DNA sequences in interphase cell nuclei is correlated to genomic distance and permits ordering of cosmids spanning 250 kilobase pairs. *Genomics*, **5**, 710–717.

TURC-CAREL, C., PHILIP, I., BERGER, M.-P., PHILIP, T. & LENOIR, G.M. (1983). Chromosomal translocations in Ewing's sarcoma. *N. Engl. J. Med.*, **309**, 497–498.

WEITH, A., MARTINSSON, T., CZIEPLUCH, C., BRUDERLEIN, S., AMLER, L.C., BERTHOLD, F. & SCHWAB, M. (1989). Neuroblastoma consensus deletion maps to 1p36.1-2. *Genes, Chromosomes & Cancer*, **1**, 159–166.

WHANG-PENG, J., TRICHE, T.J., KNUTSEN, T., MISER, J., DOUGLASS, E. & ISRAEL, M.A. (1984). Chromosomal translocation in peripheral neuroepithelioma. *N. Engl. J. Med.*, **311**, 584–585.

WILLIAMS, S.V., JONES, T.A., COTTRELL, S., ZEHETNER, G., VARESCO, L., WARD, T., THOMAS, H., LAWSON, P.A., SOLOMON, E., BODMER, W.F., FRISCHAUF, A.-M., SHEER, D. (1991). Fine mapping of probes in the adenomatous polyposis coli region of chromosome 5 by *in situ* hybridization. *Genes, Chromosomes & Cancer*, **3**, 382–389.